# Supplementary material for: Rehabilitation time has greater influences on soil mechanical composition and erodibility than does rehabilitation land type in the hilly-gully region of the Loess Plateau, China
Source: PeerJ. 2019 Nov 21;7:e8090. doi: 10.7717/peerj.8090 (PMC6875390; doi:10.7717/peerj.8090)
Supplement: Table S1 — Different lower-case letters above the bars mean significant differences among different ages within the same rehabilitation patterns (P < 0.05), * means significant differences between the natural forest and various vegetation restoration patterns at each last restoration year (P < 0.05). [file peerj-07-8090-s002.docx]

| Rehabilitation type | Rehabilitation time | Particle fractal dimension | | | | |
| --- | --- | --- | --- | --- | --- | --- |
|  |  | 0-10cm | 10-20cm | 20-30cm | 30-50cm | 50-100cm |
| Naturally revegetated grassland | 0yr | 2.711±(0.015)a | 2.713±(0.036)a | 2.707±(0.034)a | 2.705±(0.036)a | 2.695±(0.017)a |
|  | 2yr | 2.687±(0.015)ab | 2.688±(0.018)ab | 2.683±(0.018)ab | 2.689±(0.022)ab | 2.688±(0.021)a |
|  | 5yr | 2.666±(0.02)bc | 2.671±(0.025)ab | 2.671±(0.029)ab | 2.668±(0.021)ab | 2.67±(0.013)a |
|  | 8yr | 2.67±(0.027)bc | 2.681±(0.029)ab | 2.689±(0.029)ab | 2.7±(0.031)ab | 2.699±(0.04)a |
|  | 11yr | 2.664±(0.021)bc | 2.67±(0.012)ab | 2.663±(0.005)ab | 2.66±(0.012)ab | 2.655±(0.017)a |
|  | 15yr | 2.668±(0.016)bc | 2.673±(0.016)ab | 2.675±(0.022)ab | 2.679±(0.025)ab | 2.681±(0.027)a |
|  | 18yr | 2.657±(0.012)bc | 2.672±(0.014)ab | 2.667±(0.023)ab | 2.664±(0.036)ab | 2.659±(0.034)a |
|  | 26yr | 2.637±(0.018)c | 2.645±(0.03)b | 2.651±(0.027)b | 2.651±(0.036)b | 2.652±(0.033)a |
|  | 30yr | 2.662±(0.029)bc | 2.666±(0.03)b | 2.673±(0.03)ab | 2.672±(0.031)ab | 2.672±(0.03)a |
| Natural forest | ＞160yr | 2.593±(0.03)*** | 2.62±(0.018)** | 2.627±(0.021)** | 2.636±(0.018)* | 2.637±(0.022)* |
| Woodland | 0yr | 2.711±(0.015)a | 2.713±(0.036)a | 2.707±(0.034)a | 2.705±(0.036)a | 2.695±(0.017)a |
|  | 5yr | 2.682±(0.034)ab | 2.687±(0.038)a | 2.684±(0.039)a | 2.685±(0.043)a | 2.68±(0.027)a |
|  | 10yr | 2.632±(0.05)bc | 2.668±(0.021)ab | 2.664±(0.03)ab | 2.67±(0.026)ab | 2.685±(0.032)a |
|  | 20yr | 2.665±(0.004)abc | 2.674±(0.01)ab | 2.672±(0.003)ab | 2.669±(0.013)ab | 2.663±(0.014)ab |
|  | 37yr | 2.605±(0.028)c | 2.608±(0.016)b | 2.601±(0.03)b | 2.605±(0.039)b | 2.609±(0.033)b |
|  | 50yr | 2.649±(0.004)abc | 2.677±(0.03)a | 2.669±(0.03)ab | 2.665±(0.034)ab | 2.666±(0.028)ab |
| Natural forest | ＞160yr | 2.593±(0.03)* | 2.62±(0.018)** | 2.627±(0.021)* | 2.636±(0.018) | 2.637±(0.022) |
| Shrubland | 0yr | 2.711±(0.015)a | 2.713±(0.036)a | 2.707±(0.034)a | 2.705±(0.036)a | 2.695±(0.017)a |
|  | 5yr | 2.672±(0.014)b | 2.678±(0.012)ab | 2.674±(0.015)ab | 2.669±(0.01)ab | 2.661±(0.011)ab |
|  | 10yr | 2.67±(0.022)b | 2.679±(0.028)ab | 2.684±(0.026)ab | 2.674±(0.036)ab | 2.671±(0.027)ab |
|  | 20yr | 2.633±(0.011)b | 2.638±(0.011)b | 2.645±(0.013)b | 2.654±(0.012)b | 2.656±(0.012)b |
|  | 30yr | 2.644±(0.015)b | 2.667±(0.004)b | 2.674±(0.006)ab | 2.679±(0.01)ab | 2.674±(0.019)ab |
|  | 36yr | 2.653±(0.014)b | 2.657±(0.008)b | 2.648±(0.004)b | 2.644±(0.009)b | 2.645±(0.012)b |
|  | 47yr | 2.65±(0.025)b | 2.662±(0.023)b | 2.663±(0.026)b | 2.669±(0.023)ab | 2.651±(0.02)b |
| Natural forest | ＞160yr | 2.593±(0.03)* | 2.62±(0.018)** | 2.627±(0.021)* | 2.636±(0.018)* | 2.637±(0.022) |
| Orchardland | 0yr | 2.711±(0.015)a | 2.713±(0.036)a | 2.707±(0.034)a | 2.705±(0.036)a | 2.695±(0.017)ab |
|  | 5yr | 2.718±(0.023)a | 2.72±(0.018)a | 2.725±(0.031)a | 2.739±(0.027)a | 2.749±(0.016)a |
|  | 10yr | 2.716±(0.014)a | 2.72±(0.007)a | 2.722±(0.022)a | 2.723±(0.028)a | 2.729±(0.025)ab |
|  | 20yr | 2.666±(0.012)b | 2.679±(0.031)a | 2.685±(0.037)a | 2.689±(0.042)a | 2.662±(0.037)b |
| Natural forest | ＞160yr | 2.593±(0.03)** | 2.62±(0.018)** | 2.627±(0.021)** | 2.636±(0.018) | 2.637±(0.022) |

Stable1. Particle fractal dimensions of different rehabilitation type over different years
