# Supplementary material for: Rehabilitation time has greater influences on soil mechanical composition and erodibility than does rehabilitation land type in the hilly-gully region of the Loess Plateau, China
Source: PeerJ. 2019 Nov 21;7:e8090. doi: 10.7717/peerj.8090 (PMC6875390; doi:10.7717/peerj.8090)
Supplement: Table S2 — Different lower-case letters above the bars mean significant differences among different ages within the same rehabilitation patterns (P < 0.05), * means significant differences between the natural forest and various vegetation restoration patterns at each last restoration year (P < 0.05) [file peerj-07-8090-s003.docx]

| Rehabilitation type | Rehabilitation time | Micro-aggregate fractal dimension | | | | |
| --- | --- | --- | --- | --- | --- | --- |
|  |  | 0-10cm | 10-20cm | 20-30cm | 30-50cm | 50-100cm |
| Naturally revegetated grassland | 0yr | 2.6±(0.007)a | 2.6±(0.002)a | 2.588±(0.006)ab | 2.596±(0.015)ab | 2.596±(0.017)ab |
|  | 2yr | 2.602±(0.039) | 2.59±(0.044)a | 2.597±(0.052)ab | 2.596±(0.054)ab | 2.608±(0.052)ab |
|  | 5yr | 2.603±(0.053)a | 2.604±(0.057)a | 2.593±(0.058)ab | 2.599±(0.053)ab | 2.591±(0.049)ab |
|  | 8yr | 2.594±(0.021)a | 2.594±(0.022)a | 2.601±(0.03)ab | 2.616±(0.028)a | 2.618±(0.024)a |
|  | 11yr | 2.57±(0.01)a | 2.58±(0.004)a | 2.554±(0.02)b | 2.55±(0.016)b | 2.555±(0.006)b |
|  | 15yr | 2.594±(0.043)a | 2.582±(0.062)a | 2.588±(0.045)ab | 2.597±(0.042)ab | 2.59±(0.047)ab |
|  | 18yr | 2.607±(0.019)a | 2.624±(0.009)a | 2.629±(0.016)a | 2.615±(0.033)a | 2.613±(0.004)ab |
|  | 26yr | 2.614±(0.025)a | 2.599±(0.022)a | 2.607±(0.014)ab | 2.614±(0.038)a | 2.598±(0.026)ab |
|  | 30yr | 2.584±(0.025)a | 2.585±(0.02)a | 2.582±(0.02)ab | 2.588±(0.03)ab | 2.59±(0.039)ab |
| Natural forest | ＞160yr | 2.548±(0.031)* | 2.584±(0.022) | 2.58±(0.02) | 2.607±(0.021) | 2.621±(0.017) |
| Woodland | 0yr | 2.6±(0.007)a | 2.6±(0.002)a | 2.588±(0.006)a | 2.596±(0.015)ab | 2.596±(0.017)a |
|  | 5yr | 2.593±(0.042)ab | 2.569±(0.043)ab | 2.579±(0.031)a | 2.579±(0.017)ab | 2.57±(0.017)a |
|  | 10yr | 2.547±(0.022)bc | 2.525±(0.027)b | 2.507±(0.017)b | 2.524±(0.009)b | 2.546±(0.032)a |
|  | 20yr | 2.575±(0.008)abc | 2.582±(0.015)ab | 2.57±(0.014)ab | 2.558±(0.017)b | 2.549±(0.011)a |
|  | 37yr | 2.578±(0.017)abc | 2.581±(0.027)ab | 2.578±(0.038)a | 2.595±(0.039)ab | 2.603±(0.033)a |
|  | 50yr | 2.54±(0.003)c | 2.582±(0.015)ab | 2.578±(0.013)a | 2.74±(0.276)a | 2.595±(0.037)a |
| Natural forest | ＞160yr | 2.548±(0.031) | 2.584±(0.022) | 2.58±(0.02) | 2.607±(0.021) | 2.621±(0.017) |
| Shrubland | 0yr | 2.6±(0.007)a | 2.6±(0.002)ab | 2.588±(0.006)a | 2.596±(0.015)ab | 2.596±(0.017)abc |
|  | 5yr | 2.54±(0.04)ab | 2.523±(0.045)ab | 2.546±(0.023)ab | 2.531±(0.018)ab | 2.531±(0.027)bc |
|  | 10yr | 2.597±(0.059)ab | 2.592±(0.06)ab | 2.596±(0.055)a | 2.587±(0.091)ab | 2.592±(0.058)abc |
|  | 20yr | 2.574±(0.047)ab | 2.575±(0.032)ab | 2.587±(0.033)a | 2.588±(0.045)ab | 2.601±(0.04)abc |
|  | 30yr | 2.576±(0.053)ab | 2.605±(0.01)a | 2.61±(0.007)a | 2.617±(0.033)a | 2.619±(0.018)a |
|  | 36yr | 2.506±(0.057)b | 2.514±(0.045)b | 2.504±(0.034)b | 2.516±(0.011)b | 2.518±(0.031)c |
|  | 47yr | 2.603±(0.027)a | 2.609±(0.012)a | 2.617±(0.011)a | 2.616±(0.01)a | 2.607±(0.001)ab |
| Natural forest | ＞160yr | 2.548±(0.031)* | 2.584±(0.022) | 2.58±(0.02)* | 2.607±(0.021) | 2.621±(0.017) |
| Orchardland | 0yr | 2.6±(0.007)b | 2.6±(0.002)b | 2.588±(0.006)b | 2.596±(0.015)a | 2.596±(0.017)b |
|  | 5yr | 2.658±(0.011)a | 2.653±(0.01)a | 2.648±(0.004)a | 2.651±(0.024)a | 2.663±(0.012)a |
|  | 10yr | 2.643±(0.006)ab | 2.627±(0.011)ab | 2.62±(0.022)ab | 2.621±(0.031)a | 2.623±(0.017)ab |
|  | 20yr | 2.632±(0.008)ab | 2.648±(0.024)a | 2.648±(0.031)a | 2.656±(0.038)a | 2.622±(0.018)ab |
| Natural forest | ＞160yr | 2.548±(0.031)*** | 2.584±(0.022)*** | 2.58±(0.02)*** | 2.607±(0.021)* | 2.621±(0.017) |

Stable2. Micro-aggregate fractal dimensions of different rehabilitation type over different years
