# Supplementary material for: Rehabilitation time has greater influences on soil mechanical composition and erodibility than does rehabilitation land type in the hilly-gully region of the Loess Plateau, China
Source: PeerJ. 2019 Nov 21;7:e8090. doi: 10.7717/peerj.8090 (PMC6875390; doi:10.7717/peerj.8090)
Supplement: Table S3 — Different lower-case letters above the bars mean significant differences among different ages within the same rehabilitation patterns (P < 0.05), * means significant differences between the natural forest and various vegetation restoration patterns at each last restoration year (P < 0.05) [file peerj-07-8090-s004.docx]

| Rehabilitation type | Rehabilitation time | Erodibility | | | | |
| --- | --- | --- | --- | --- | --- | --- |
|  |  | 0-10cm | 10-20cm | 20-30cm | 30-50cm | 50-100cm |
| Naturally revegetated grassland | 0yr | 0.421±(0.011)a | 0.42±(0.012)ab | 0.42±(0.012)ab | 0.417±(0.009)a | 0.419±(0.008)a |
|  | 2yr | 0.414±(0.011)a | 0.415±(0.013)ab | 0.413±(0.013)ab | 0.416±(0.018)a | 0.419±(0.016)a |
|  | 5yr | 0.404±(0.012)ab | 0.409±(0.015)ab | 0.412±(0.014)ab | 0.411±(0.011)a | 0.413±(0.006)a |
|  | 8yr | 0.418±(0.012)a | 0.421±(0.015)ab | 0.422±(0.016)ab | 0.425±(0.016)a | 0.424±(0.018)a |
|  | 11yr | 0.385±(0.022)b | 0.392±(0.014)b | 0.394±(0.016)b | 0.397±(0.016)a | 0.4±(0.015)a |
|  | 15yr | 0.397±(0.023)ab | 0.401±(0.025)ab | 0.408±(0.024)ab | 0.41±(0.027)a | 0.412±(0.026)a |
|  | 18yr | 0.418±(0.002)a | 0.425±(0.01)a | 0.426±(0.012)a | 0.424±(0.015)a | 0.419±(0.019)a |
|  | 26yr | 0.41±(0.013)ab | 0.419±(0.02)ab | 0.423±(0.019)ab | 0.422±(0.019)a | 0.425±(0.022)a |
|  | 30yr | 0.398±(0.011)ab | 0.406±(0.013)ab | 0.411±(0.018)ab | 0.414±(0.017)a | 0.417±(0.021)a |
| Natural forest | ＞160yr | 0.326±(0.045)*** | 0.382±(0.039) | 0.402±(0.034) | 0.426±(0.008) | 0.436±(0.007) |
| Woodland | 0yr | 0.421±(0.011)a | 0.42±(0.012)a | 0.42±(0.012)a | 0.417±(0.009)a | 0.419±(0.008)ab |
|  | 5yr | 0.395±(0.016)ab | 0.4±(0.018)ab | 0.4±(0.015)ab | 0.405±(0.021)ab | 0.405±(0.015)ab |
|  | 10yr | 0.387±(0.026)ab | 0.418±(0.01)a | 0.422±(0.012)a | 0.419±(0.016)a | 0.418±(0.011)ab |
|  | 20yr | 0.392±(0.011)ab | 0.403±(0.005)ab | 0.406±(0.005)ab | 0.406±(0.007)ab | 0.411±(0.007)ab |
|  | 37yr | 0.373±(0.039)bc | 0.413±(0.021)ab | 0.416±(0.017)a | 0.418±(0.016)a | 0.423±(0.016)a |
|  | 50yr | 0.342±(0.029)c | 0.387±(0.001)b | 0.385±(0.004)b | 0.383±(0.001)b | 0.394±(0.007)b |
| Natural forest | ＞160yr | 0.326±(0.045) | 0.382±(0.039) | 0.402±(0.034) | 0.426±(0.008)*** | 0.436±(0.007)*** |
| Shrub land | 0yr | 0.421±(0.011)a | 0.42±(0.012)a | 0.42±(0.012)a | 0.417±(0.009)a | 0.419±(0.008)a |
|  | 5yr | 0.388±(0.003)ab | 0.393±(0.002)b | 0.397±(0.009)a | 0.402±(0.012)a | 0.4±(0.008)a |
|  | 10yr | 0.401±(0.02)ab | 0.407±(0.017)ab | 0.408±(0.016)a | 0.409±(0.02)a | 0.419±(0.023)a |
|  | 20yr | 0.393±(0.029)ab | 0.406±(0.018)ab | 0.411±(0.025)a | 0.416±(0.023)a | 0.422±(0.026)a |
|  | 30yr | 0.364±(0.025)b | 0.406±(0.009)ab | 0.415±(0.009)a | 0.418±(0.009)a | 0.427±(0.006)a |
|  | 36yr | 0.364±(0.041)b | 0.399±(0.003)ab | 0.402±(0.004)a | 0.405±(0.007)a | 0.409±(0.007)a |
|  | 47yr | 0.405±(0.015)ab | 0.415±(0.008)ab | 0.421±(0.008)a | 0.424±(0.006)a | 0.43±(0.011)a |
| Natural forest | ＞160yr | 0.326±(0.045)* | 0.382±(0.039) | 0.402±(0.034) | 0.426±(0.008) | 0.436±(0.007) |
| Orchard land | 0yr | 0.421±(0.011)a | 0.42±(0.012)a | 0.42±(0.012)a | 0.417±(0.009)a | 0.419±(0.008)a |
|  | 5yr | 0.421±(0.007)a | 0.421±(0.004)a | 0.426±(0.006)a | 0.428±(0.011)a | 0.433±(0.011)a |
|  | 10yr | 0.409±(0.008)a | 0.415±(0.005)a | 0.418±(0.005)a | 0.416±(0.006)a | 0.42±(0.006)a |
|  | 20yr | 0.416±(0.01)a | 0.428±(0.009)a | 0.431±(0.009)a | 0.433±(0.009)a | 0.428±(0.014)a |
| Natural forest | ＞160yr | 0.326±(0.045)** | 0.382±(0.039) | 0.402±(0.034) | 0.426±(0.008) | 0.436±(0.007) |

Stable3. Erodibilitys of different rehabilitation type over different years
