# Supplementary figures and images for: Rehabilitation time has greater influences on soil mechanical composition and erodibility than does rehabilitation land type in the hilly-gully region of the Loess Plateau, China
Source: PeerJ. 2019 Nov 21;7:e8090. doi: 10.7717/peerj.8090 (PMC6875390; doi:10.7717/peerj.8090)

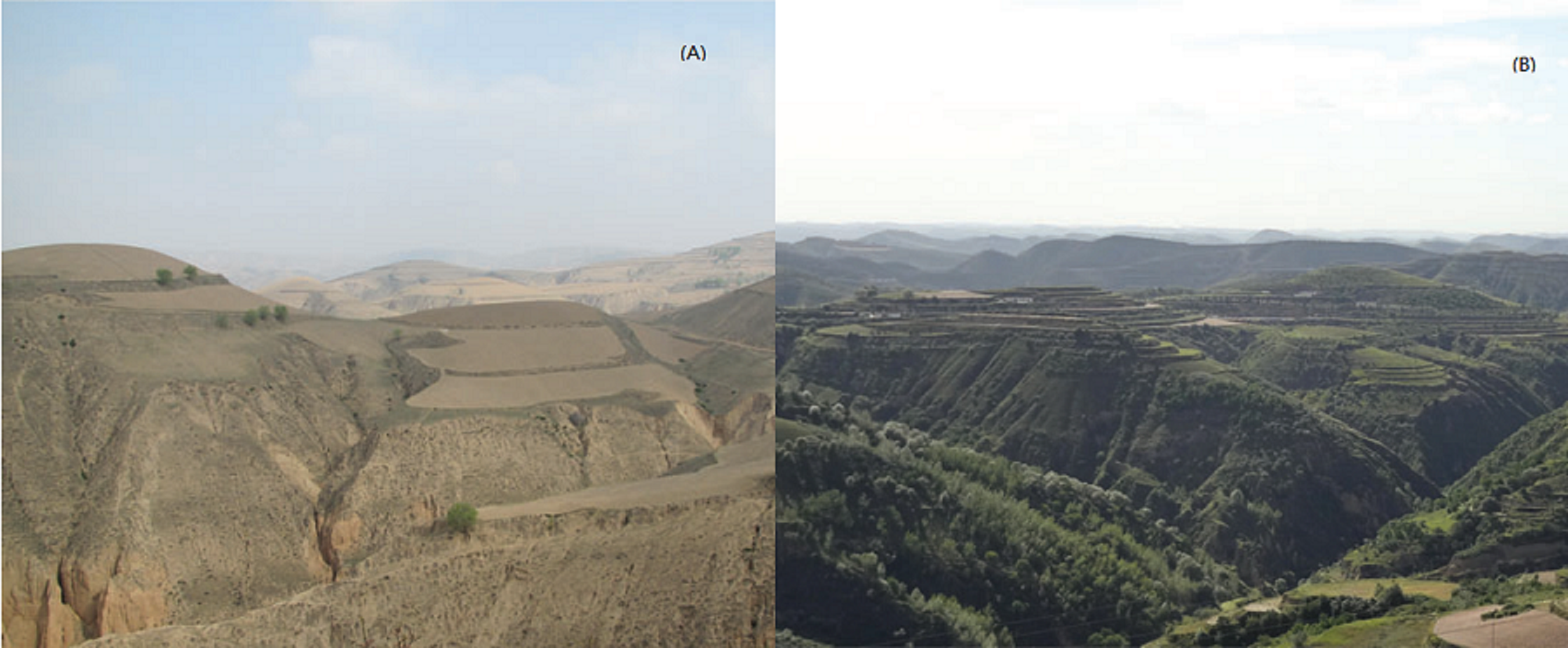

Supplement: Figure S1 — Before (A) and after (B) revegetation on the hilly Loess Plateau. [file peerj-07-8090-s005.png]
